# Role of Interfacial Coherency on Creep Behavior of FCC/BCC High-Entropy Alloy Multilayers

**DOI:** 10.3390/ma19051028

**Published:** 2026-03-07

**Authors:** Junwei Zhou, Jinrui Tang, Zhien Ning, Xiaofeng Yang, Min Gu, Chundi Fan, Junming Chen, Zhaoming Yang, Guoqiang Zeng

**Affiliations:** 1 College of Nuclear Technology and Automation Engineering, Chengdu University of Technology, Chengdu 610059, China; zhoujunwei@cdut.edu.cn (J.Z.);; 2 The First Sub-Institute, Nuclear Power Institute of China, Chengdu 610041, China; 3 Applied Nuclear Technology in Geosciences Key Laboratory of Sichuan Province, Chengdu University of Technology, Chengdu 610059, China

**Keywords:** high-entropy alloys, multilayers, heterogeneous interfaces, creep mechanism, nanoindentation

## Abstract

High-entropy alloy (HEA) multilayers represent a promising class of advanced coating materials due to their superior mechanical properties, corrosion resistance, and irradiation tolerance. However, the specific role of interface coherency on the creep behavior of HEA multilayers remains unclear. In this work, FCC/BCC Al-Cr-Fe-Ni HEA multilayers with different coherency were prepared by precisely controlling the modulated period (λ) via RF magnetron sputtering. Their room-temperature creep properties were systematically investigated through nanoindentation under different loading rates. The results reveal a strong dependence of creep resistance and deformation mechanisms on the interface coherency. HEA multilayers with semicoherent interfaces (λ = 16 nm) exhibit the highest creep resistance, where creep is primarily mediated by atomic diffusion or interface slip. In contrast, samples dominated by coherent interfaces or grain boundaries (λ = 8, 32, and 80 nm) demonstrate dislocation slip-dominated creep. This work elucidates how interfacial coherency dictates the transition between diffusion-mediated and dislocation-mediated creep mechanisms in HEA multilayers, providing critical insights for the design of next-generation creep-resistant nanostructured coatings.

## 1. Introduction

Surface engineering through protective coatings is a cornerstone technology for enhancing the durability and longevity of critical components across aerospace, energy, industries, etc. [1,2,3,4]. These coatings are perpetually subjected to severe degradation behaviors under applied environments, including wear, corrosion, creep deformation and irradiation damage, thus posing significant risks to safety and operational efficiency. Consequently, the development of next-generation coating materials that offer superior and multifunctional performance, combining high strength, thermal stability, wear resistance, irradiation tolerance, and so on, is not merely an academic pursuit but an industrial imperative.

High-entropy alloys (HEAs), characterized by multiple principal elements, have been introduced as a novel concept in materials science [5,6]. HEAs are defined by four core effects, high entropy, severe lattice distortion, sluggish diffusion, and the cocktail effects, which collectively contribute to exceptional mechanical properties, remarkable thermal stability, excellent corrosion resistance and irradiation tolerance [7,8,9]. When this novel component design is combined with a nanoscale multilayer architecture strategy, a new class of HEA multilayer coatings emerged [10,11]. HEA multilayers synergistically harness the intrinsic strengthening of HEAs with extrinsic strengthening from interfaces [12,13]. The multilayer structure enables the precise tailoring of microstructural features such as the interfacial density and interfacial structure, which are key factors in controlling mechanical properties [13]. This heterogeneous structure design can induce non-uniform deformation, leading to significant non-uniform deformation-induced strengthening and an enhanced strain hardening capacity, thereby achieving an optimal balance between strength and ductility [13,14]. This makes HEA multilayers a prime candidate material for overcoming the limitations of traditional coating systems.

For the coatings used in continuous load applications, resistance to plastic deformation over time (also known as creep) is a critical performance metric. The creep behavior of metallic multilayer materials is related to the modulation period and interfacial structure, which together determine dislocation dynamics under sustained stress [15,16,17]. In multilayers, the modulation period (λ) serves as a critical length scale that determines whether deformation is dominated by confined layer slip (CLS) or interface-mediated mechanisms [15]. When λ is reduced to a few nanometers, the interface density increases dramatically, leading to a transition from bulk-like dislocation glide to interface-controlled creep, where the interfaces act as both barriers and sources for dislocations [15,16]. From an interface perspective, the structure of the interface (typically coherent and semicoherent interfaces within metallic multilayers) plays a crucial role in determining the creep behavior and mechanisms of multilayers, primarily by regulating their interactions with the plastic deformation carrier. Extensive studies on the deformation behavior of conventional metallic multilayers have established a consistent consensus; coherent interfaces provide low-energy pathways for dislocation transmission and enhancing the ductility of materials, whereas semicoherent interfaces tend to obstruct the slip of dislocations, resulting in an increased strength of materials [15,16,17].

Although the deformation mechanics of conventional multilayer materials provide a fundamental framework, the behavior of HEA multilayer materials is expected to be different and more complex. The inherent characteristics of HEAs, such as severe lattice distortion, compositional complexity, and short-range ordered structures, give rise to unique deformation mechanisms, including enhanced solid solution strengthening, altered dislocation core structures, pronounced plane slip and nanoscale twinning [9,18,19]. The introduction of high-density heterogeneous interfaces in multilayer architectures further exacerbates this complexity. Preliminary evidence suggests that these interactions are pivotal for the deformation behaviors of multilayers. For example, in FCC/BCC HEA multilayers, semicoherent interfaces were found to accumulate dislocations under creep conditions and result in amorphization, whereas coherent interfaces did not [20]. This indicates that the interface structure may govern the primary creep mechanism in HEA systems, whether through conventional dislocation blocking, the activation of alternative deformation modes, or the triggering of interface-based phase transformations. However, the correlation mechanism between the heterogeneous interface structures and the creep behavior of HEA multilayers remains poorly understood, which severely impedes the design of advanced creep-resistant coatings.

In this work, to investigate the influence of interfacial coherency on the creep behavior of HEA multilayers, FCC/BCC Al-Cr-Fe-Ni HEA multilayers with different coherency of heterogeneous interfaces were prepared by controlling modulation periods using the RF magnetron sputtering method. The creep behavior of HEA multilayers under varying loading rates was analyzed by using nanoindentation. The results show that the heterogeneous interface structure significantly alters the creep mechanism of FCC/BCC HEA multilayers. A semicoherent interface promotes atomic diffusion or interface slip-type creep, whereas a coherent interface and grain boundary are beneficial to dislocation slip-dominated creep.

## 2. Materials and Methods

FCC/BCC high-entropy alloy (HEA) multilayers were deposited via an RF magnetron sputtering system. The sputtering targets consisted of a single-phase FCC Al0.2CrFeNi HEA plate (99.99% purity) and a pure Al plate (99.999% purity). The deposition chamber was evacuated to a base pressure of approximately 5 × 10^−4^ Pa prior to deposition. Argon gas was then introduced at a flow rate of 100 sccm, establishing a working pressure of 0.3 Pa. During the fabrication of multilayers with different individual layer thicknesses (also known as the modulated period, λ = 8, 16, 32, and 80 nm), the sputtering power for the Al0.2CrFeNi HEA target was maintained at 150 W, while the power for the Al target alternated between 0 W and 30 W. Each sample was deposited for 1 h, yielding FCC/BCC Al-Cr-Fe-Ni HEA multilayers with a total thickness of approximately 1 μm. For convenience, the as-prepared multilayers were denoted as “Mλ”, in which “M” and “λ” represent the multilayers and the modulated period, respectively.

In our prior study, the critical individual layer thickness (*h_c_*) for the transition from semicoherent to coherent interfaces in this HEA multilayer was determined to be approximately 11 nm [20]. The interface structure was examined using X-ray diffraction (XRD) and transmission electron microscopy (TEM) [20]. In the XRD patterns, distinct diffraction peaks corresponding to the (111) plane of the FCC phase and the (110) plane of the BCC phase were observed in samples with λ ≥ 16, whereas these peaks completely overlapped in sample M8. Inverse fast Fourier transform (IFFT) images of interface regions revealed semicoherent interfaces containing misfit dislocations in sample M16, and coherent interfaces with continuous atomic planes in sample M8. Furthermore, in samples M80 and M32, grain boundaries constitute the predominant interfaces, since λ exceeds the grain diameter of about 20 nm, as obtained from XRD analysis.

The nanoindentation tests at room temperature were performed using an nanoindenter (Anton Paar, NHT^3^, Graz Austria), whose load resolution and displacement resolution are 20 nN and 0.01 nm, respectively. To control the indentation depth between 1/10 and 1/7 of the coating thickness and prevent the mechanical property data from being influenced by the sample surface or substrate material, the maximum load was set to 5 mN. To investigate the mechanical response of samples under different loading rates, loading rates of 1, 0.1 and 0.01 mN/s, with a hold time of 100 s and an unloading time of 20 s, were adopted. Displacement–dwell time and force–indent displacement curves were recorded during indentation. To minimize potential errors, at least five independent indentations were performed on each sample. To prevent interference between indentations, the spacing between adjacent indent sites was set at 30 μm. Typical force–displacement curves of each sample were presented in this work. Normalized displacement–dwell time curves were statistically averaged to reveal the relationship between creep displacement and dwell time. Moreover, the time-dependent creep rate and creep stress exponent were calculated from the averaged normalized displacement–dwell time curves.

## 3. Results and Discussion

**Figure 1:**
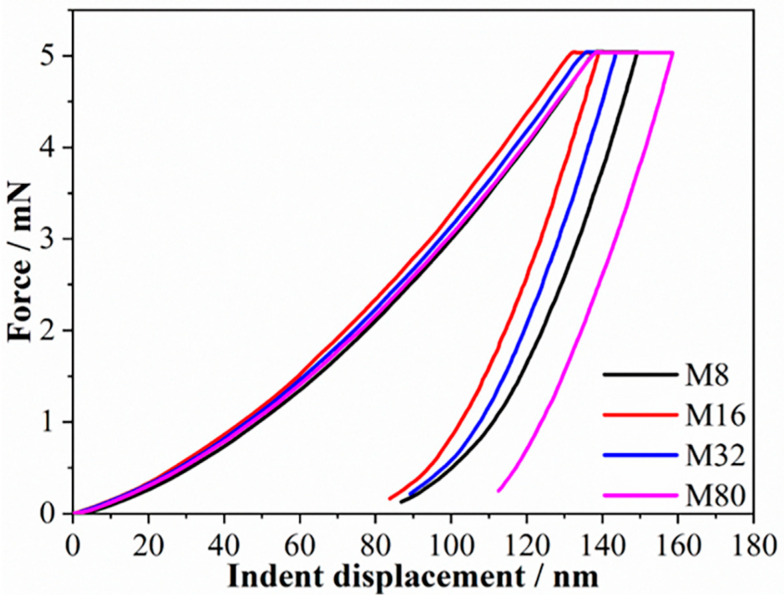
The force–indent displacement curves of the samples.

**Figure 2:**
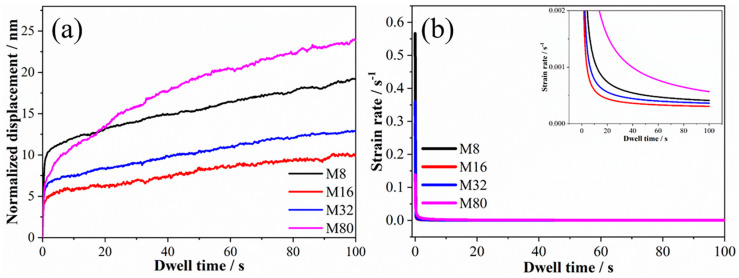
The normalized displacement–dwell time curves (**a**) and strain rate–dwell time curves (**b**) of the samples.

The force–indent displacement curves tested at a loading rate of 1 mN/s are shown in Figure 1. The indentation depth of all samples is between 100 and 160 nm, corresponding to 1/10 to 1/7 of the coatings’ thickness, indicating that the influence of the sample surface and Si substrate on the measured mechanical properties of the coatings is negligible. Moreover, no “step-like” discontinuity or displacement (known as “pop-in”) events were observed. However, the curves exhibit a significant dispersion. The indent depth decreases initially and then increases as λ reduces, and sample M16 exhibits the smallest indentation depth. The creep displacement–dwell time curves are shown in Figure 2a. The creep displacement of the samples increased with increasing holding time. For all samples, the increase rate in creep displacement was fastest at the early stage of holding. Moreover, the creep displacement at the early stage of holding varied with λ, and the samples M8 and M16 exhibited the maximum and minimum creep displacements, respectively. The strain rate was investigated to elucidate the creep behavior, as shown in Figure 2b. As the holding time increases, the creep rate of the sample rapidly decreases from a large value (about 0.1–0.6 s^−1^) to an approximately stable and smaller value (the order of 10^−4^ s^−1^) within a very short time, indicating the onset of steady-state creep. The steady-state creep rate decreased from 5.7 × 10^−4^ s^−1^ to 3.1 × 10^−4^ s^−1^ when λ decreased from 80 nm to 16 nm. However, when λ is further reduced to 8 nm, the steady-state creep rate increases to 4.1 × 10^−4^ s^−1^.

**Figure 3:**
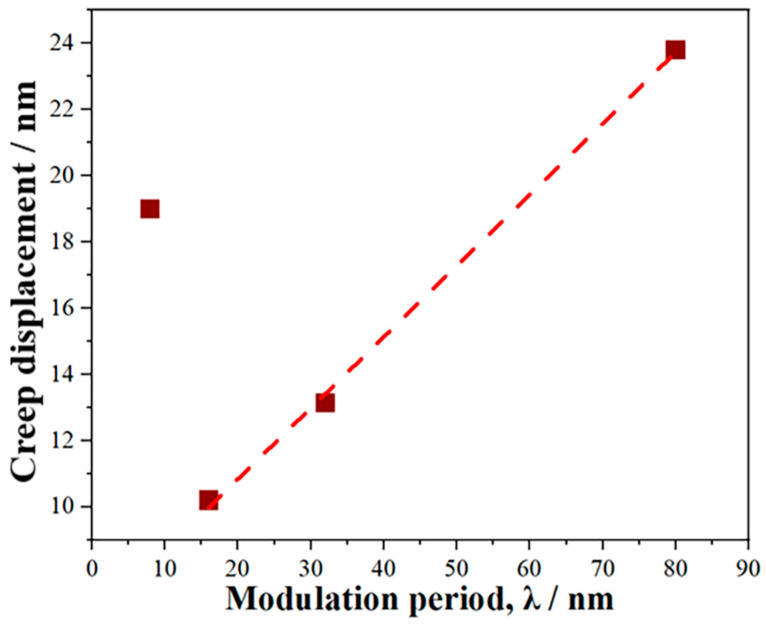
The relationship between creep displacement and modulation periods of the samples.

The relationship between creep displacements and λ is plotted in Figure 3. As λ decreased, the creep displacement initially decreased and then increased, and the creep displacement of sample M16 is the smallest. These results indicate that the creep resistance first enhanced and then deteriorated, and sample M16 has the strongest creep resistance. The creep displacement decreases linearly from 24 nm to 10 nm when λ decreases from 80 nm to 16 nm. Previous studies on the plastic deformation mechanism of multilayer films have demonstrated that dislocation motion is inhibited by interfaces, leading to dislocation pile-up at the interfaces and enhancing the material’s resistance to plastic deformation. [20,21]. The relationship between the material strength and λ can be described using the Hall–Petch relationship, and the λ-dependent creep rate can be expressed as follows [22]:

(1) $$\dot{\varepsilon }\left(t\right)\;=\;\mathrm{Bexp}[-\frac{Q}{\mathrm{RT}}\;+\;C\left(\sigma_{S}-\sigma_{0}-k\lambda^{-\frac{1}{2}}\right)]$$

where *B*, *C*, and *k* are constants, $\sigma_{S}$ is the flow stress, and $\sigma_{0}$ is the lattice friction stress. Equation (1) reveals that the creep rate increases with increasing λ, consistent with the results in Figure 2b. Therefore, the creep displacement decreases with reducing λ. However, when λ decreased to 8 nm, the creep displacement unexpectedly increased to 19 nm, indicating a weaker obstruction of dislocation movement for the coherent heterogeneous interface in sample M8. These phenomena suggest that the creep behavior of the Al-Cr-Fe-Ni FCC/BCC high-entropy alloy multilayer coatings is strongly dependent on λ and the hetero-interface structure.

**Figure 4:**
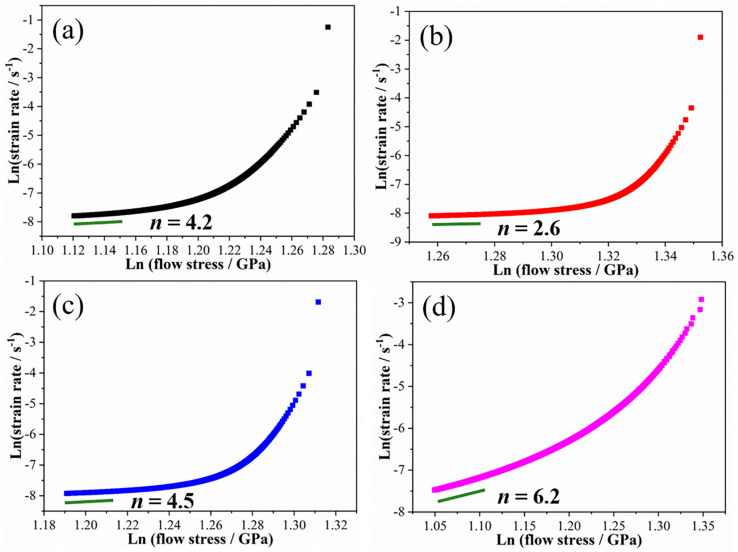
The *ln*(strain rate)–*ln*(flow stress) curves and stress exponent (*n*) of the samples M8 (**a**), M16 (**b**), M32 (**c**) and M80 (**d**).

To elucidate the creep mechanisms of FCC/BCC high-entropy alloy (HEA) multilayers, the creep stress exponent (*n*) was investigated by linear fitting the strain rate–creep stress relationship curve at the steady-state creep stage; the results are shown in Figure 4a–d. The variation trend of *n* with λ is similar to that of the creep displacement and steady-state creep rate: *n* first decreases and then increases as λ decreases, with sample M16 reaching the minimum value. The value of *n* reflects the creep mechanism of the material. *n* = 1 suggests that the creep of the material is controlled by atomic diffusion, i.e., the diffusion creep mechanism; *n* = 2 indicates that the creep of the material is controlled by grain boundary slip, i.e., the grain boundary slip mechanism; and *n* > 3 indicates that the creep of the material is controlled by dislocation slip or climb, i.e., the dislocation mechanism [23,24]. Note that the *n* obtained in the nanoindentation test is usually slightly higher than the *n* obtained in the uniaxial tensile test, but it is an equally important parameter for analyzing the creep mechanism of materials [23,24,25]. In this work, the *n* of sample M16 is 2.6, indicating that its creep deformation is dominated by atomic diffusion or grain boundary slip, while the *n* of the other samples is greater than 3, indicating that their creep deformation is dominated by dislocation slip or climb. The diversity in the creep mechanism of the samples may be attributed to the differences in the dominant interfaces in the samples because the interfaces play a crucial role in dislocation motion and atomic migration [23]. Grain boundaries are the dominant interfaces in samples M80 and M32, whereas the semicoherent and coherent interfaces between layers become dominant in samples M16 and M8, respectively [20,26,27]. These results suggest that coherent heterogeneous interfaces and grain boundaries are conducive to dislocation-type creep in HEA multilayers, while semicoherent heterogeneous interfaces promote diffusion-type creep or interface slip-type creep in HEA multilayers.

**Figure 5:**
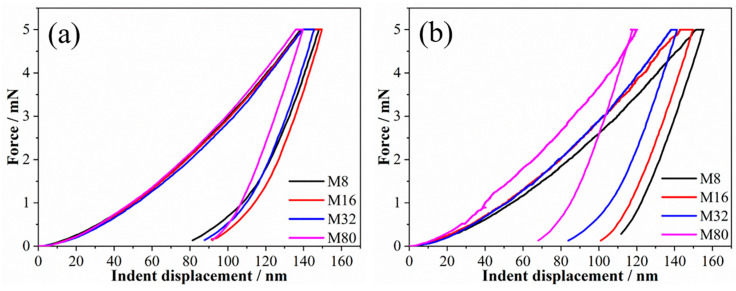
The force–indent displacement curves of the samples in loading rates of 0.1 mN/s (**a**) and 0.01 mN/s (**b**).

To investigate the influence of the loading rate on the creep behavior and creep resistance of HEA multilayers, nanoindentation creep tests were conducted at room temperature using loading rates of 0.1 mN/s and 0.01 mN/s; the obtained force–indent displacement (P-h) curves are shown in Figure 5. The indent depths are between 1/10 and 1/7 of the thickness of multilayers. No obvious “pop-in” event was observed in the p-h curve tested at 0.1 mN/s, but a distinct “pop-in” event was observed at 0.01 mN/s. Generally, the “pop-in” event in nanoindentation experiments is closely related to the dislocation nucleation and plastic deformation of the material. At a larger loading rate, the instantaneous deformation is suppressed by continuous plastic deformation, and thus no well-developed pop-ins are observed [28,29]. The indentation depth of the sample exhibits a distinct loading rate dependency, as shown in Figure 1 and Figure 5. For sample M8, the indentation depths at the loading rates of 1 mN/s and 0.1 mN/s were comparable (~149 nm) and increased to ~155 nm as the loading rate decreased to 0.01 mN/s. For sample M16, the indentation depth at a loading rate of 1 mN/s was ~138 nm, and that increased to ~150 nm as the loading rate decreased to 0.1 mN/s and 0.01 mN/s. For sample M32, the indentation depth varied slightly, increasing from ~143 nm to ~145 nm as the loading rate decreased from 1 mN/s to 0.1 mN/s, and decreasing to ~141 nm as the loading rate continued to decrease to 0.01 mN/s. For sample M80, the indentation depth decreased demonstrably, decreasing from ~158 nm to ~139 nm as the loading rate decreased from 1 mN/s to 0.1 mN/s, then decreased to ~116 nm, and a noticeable rebound phenomenon was observed as the loading rate decreased to 0.01 mN/s. In general, the indentation depth of the M8 and M16 samples increased with the decrease in loading rate, while the M32 sample was changed slightly and the M80 sample decreased significantly. It is noteworthy that the change trend of the indentation depth with λ altered significantly. The indentation depth decreased first and then increased as λ decreased at a loading rate of 1 mN/s, and sample M16 had the smallest indent depth. However, the indentation depth increased as λ decreased at loading rates of 0.1 mN/s and 0.01 mN/s, and the indentation depths of samples M8 and M16 were comparable.

**Figure 6:**
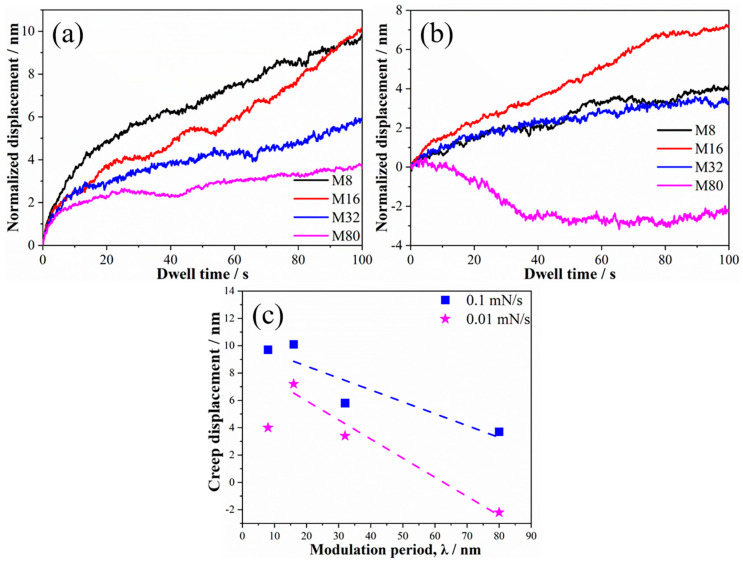
The normalized displacement–dwell time curves of the samples in loading rates of 0.1 mN/s (**a**) and 0.01 mN/s (**b**); the relationship between creep displacement and modulation period (**c**).

As shown in Figure 2a and Figure 6a,b, the creep displacement of all samples decreased with the reduced loading rate, which is mainly induced by the sharply decrement of the creep displacement in the transient creep stage with the reduced loading rate. This phenomenon is attributed to the lower accumulated elastic strain energy at low loading rates due to the extended time required to reach maximum load. The creep displacement of all samples increased with prolonged holding time at a loading rate of 0.1 mN/s, which is similar to the trend for creep displacement at a loading rate of 1 mN/s. The creep displacement of samples M8, M16 and M32 increased with extended load-holding time, but sample M80 exhibited a negative creep displacement, indicating that plastic rebound occurred for sample M80 during the holding stage. It is noteworthy that the trend of creep displacement with λ (Figure 6c) changed significantly compared to that tested at 1 mN/s (Figure 3). At a loading rate of 0.1 mN/s, the creep displacement of the samples increased as λ decreased, with samples M16 and M8 exhibiting similar creep displacements. At a loading rate of 0.01 mN/s, the creep displacement of the samples first increased and then decreased as λ decreased, with sample M16 exhibiting the largest creep displacement.

**Figure 7:**
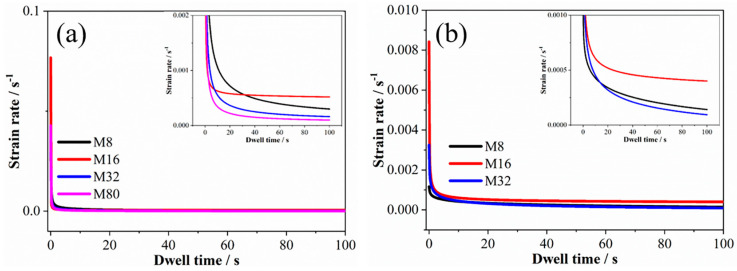
The strain rate–dwell time curves of the samples in loading rates of 0.1 mN/s (**a**) and 0.01 mN/s (**b**).

Figure 7 shows the relationship curve between the creep strain rate and the dwell time. The creep deformation of all samples comprises two stages: the transient creep stage and the steady-state creep stage. Figure 7a,b show that the strain rates during the transient creep phase for samples loaded at 0.1 mN/s and 0.01 mN/s were 0.03 to 0.08 s^−1^ and 0.001 to 0.008 s^−1^, respectively. The transient creep strain rate exhibited an order-of-magnitude decrease as the loading rate decreased. For the 0.1 mN/s loaded samples, the steady-state creep rate increased from 9.8 × 10^−4^ s^−1^ to 5.2 × 10^−4^ s^−1^ as λ decreased from 80 nm to 16 nm and increased to 3.0 × 10^−4^ s^−1^ as λ further decreased to 8 nm (see Figure 7a). For the 0.01 mN/s loading samples, the steady-state creep rate increased from 9.4 × 10^−5^ s^−1^ to 4.0 × 10^−4^ s^−1^ as the modulation period decreased from 32 nm to 16 nm and increased to 1.4 × 10^−4^ s^−1^ as λ further decreased to 8 nm (see Figure 7b). Compared to the steady-state creep rates of the 1 mN/s loading sample, that of the 0.1 mN/s and 0.01 mN/s loading samples exhibited opposite trends with respect to λ. It is worth noting that the steady-state creep strain rates of samples M80, M32, and M8 decreased significantly as the loading rate decreased, while that of sample M8 was relatively comparable.

**Figure 8:**
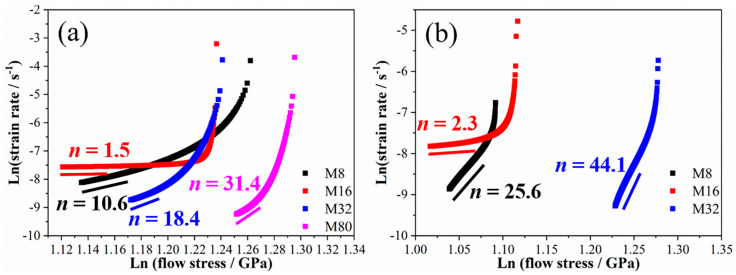
The *ln*(strain rate)–*ln*(flow stress) curves and stress exponent (*n*) of the samples at loading rates of 0.1 mN/s (**a**) and 0.01 mN/s (**b**).

The *ln*(strain rate)–*ln*(flow stress) relationship curves and the obtained stress exponent (*n*) are shown in Figure 8. At a loading rate of 0.1 mN/s, *n* decreased from 31.4 to 1.5 as λ was decreased from 80 nm to 16 nm, and then increased to 10.6 as λ was decreased to 8 nm (see Figure 8a). At a loading rate of 0.01 mN/s, *n* decreased from 44.1 (M32) to 2.3 (M16) and then increased to 25.6 (M8) with the decreasing λ (see Figure 8b). Notably, samples M80, M32, and M8 exhibited relatively large n values at low loading rates. This is attributed to the reduced three-dimensional stress concentration at low loading rates, the extended loading time facilitating the dynamic recovery of dislocations, and the earlier onset of the steady-state creep stage. The creep stress exponent exhibits a consistent variation with λ at different loading rates, indicating that, although the creep displacement and creep strain rate changed under different loading rates, the trend of the sample’s creep resistance with λ remains unchanged. Specifically, the sample’s creep resistance first increases and then decreases as λ decreases, with sample M16 exhibiting the strongest creep resistance. The creep stress exponents for samples M80, M32, and M8 are larger than 3 and increase with the decreasing loading rate, indicating that their creep is dominated by dislocation slip. The creep stress exponents for sample M8 are less than 3 and insensitive to the loading rate, suggesting that its creep is primarily driven by atomic diffusion or interface slip.

The most innovative finding of this work is that the creep behavior and mechanisms of FCC/BCC HEA multilayers are dependent on their interfacial structures. Creep is primarily driven by dislocation slip or climb for samples M80, M32, and M8, whose dominate interfaces are the grain boundaries or coherent heterogenous interfaces, whereas creep is primarily driven by atomic diffusion or interface slip for sample M16, whose dominate interface is semicoherent heterogeneous interface.

In our previous work, the interaction process between dislocations and heterogeneous interfaces and the evolution of stress distribution in coherent and semicoherent samples during deformation were investigated using molecular dynamics (MD) simulations and HRTEM [20]. The results have shown that the semicoherent heterogeneous interface strongly impedes dislocation slip and confines dislocations within the FCC layer, whereas the coherent interface is transparent to dislocation motion. In the coherent sample, dislocation slip creep occurs under applied stress due to the continuous slip of dislocations across the coherent hetero-interface between the FCC and BCC phases. In contrast, the creep behavior of the semicoherent sample is quite different because of the strong interaction between slipping dislocations and the semicoherent heterogeneous interface. The dislocation pileups at the semicoherent interface build a localized ultrahigh stress at the interface, which leads to the formation of an amorphous phase at the interface and its subsequent growth with increasing strain [20]. Under applied stress, the newly formed crystal–amorphous interface has significantly reduced atomic migration barriers and thus facilitates atomic diffusion and interface slip because of the high interfacial energy, abundant diffusion pathways provided by free volume and structural disorder, and stress gradient-driven defects and localized amorphization [30,31,32]. On the one hand, the crystal–amorphous interface possesses a higher interfacial energy and greater free volume, which significantly reduces the activation energy for atomic jumps, thus resulting in a higher atomic diffusion rate at the interface [33]. On the other hand, the amorphous phase lacks long-range order in the atomic arrangement, containing numerous vacancies, dislocations, and locally relaxed regions. These defects form a continuous “diffusion network”, enabling atoms to migrate near interfaces with low energy barriers [34]. Therefore, the interplay between dislocation blockage at semicoherent interfaces and subsequent amorphous phase formation establishes a dual-stage deformation–resistance mechanism. Initially, the semicoherent interface acts as a potent barrier to dislocation slip, promoting strain hardening via dislocation accumulation and localized stress concentration. Subsequently, the stress-induced amorphous region facilitates interfacial diffusion and slip. This process effectively redistributes stress and dissipates strain energy through viscous flow and defect-mediated atomic rearrangement. This sequential process, which transitions from dislocation blocking to stress-driven amorphization and interfacial diffusion, enhances the creep resistance of multilayer coatings. It achieves this by suppressing dislocation-based plasticity in the crystalline layers while activating alternative, rate-limited deformation modes along the crystal–amorphous interfaces. Consequently, the overall creep performance is optimized through a synergistic combination of interfacial strengthening and stress relief via controlled atomic diffusion at heterogeneous interfaces.

## 4. Conclusions

This work demonstrates that the interfacial coherency (i.e., coherent vs. semicoherent) plays a decisive role in governing the creep behavior and underlying mechanisms of FCC/BCC Al-Cr-Fe-Ni HEA multilayers. The multilayer with semicoherent interfaces (λ = 16 nm) exhibits the highest creep resistance, while deformation is primarily accommodated by atomic diffusion or interface slip. In contrast, multilayers dominated by coherent interfaces or grain boundaries (λ = 8, 32, and 80 nm) display creep behavior controlled by dislocation slip. This distinct mechanistic divergence is attributed to the fundamentally different interactions between dislocations and interfaces. Semicoherent interfaces strongly impede dislocation glide, leading to stress concentration, subsequent interface amorphization, and the activation of diffusion-mediated creep. Conversely, coherent interfaces allow for more facile dislocation transmission, favoring dislocation-mediated creep. These findings elucidate how tailoring interfacial coherency in HEA multilayers can strategically transition the dominant creep mechanism from dislocation-based to diffusion-controlled processes, providing a critical design principle for engineering advanced nanostructured coatings with superior creep resistance for demanding load-bearing applications.

## Data Availability

The original contributions presented in this study are included in the article; further inquiries can be directed to the corresponding authors.
